# A survey on perioperative antibiotic use for minimally invasive coronary artery bypass grafting

**DOI:** 10.1007/s11748-025-02136-z

**Published:** 2025-03-09

**Authors:** Xiuxiu Zhang, Chaohua Wang, Huanjun Yu, Yichang Song, Yingxue He, Tiantong Zhao, Tingting Liu, Xinyan Liu, Dapeng Yu

**Affiliations:** 1 Department of Cardiovascular Surgery, Dong E Hospital, Liaocheng, 252200 Shandong China; 2 Department of Pharmacy, Dong E Hospital, Liaocheng, 252200 Shandong China; 3 Department of Critical Care Medicine, Dong E Hospital, Liaocheng, 252200 Shandong China

**Keywords:** Minimally invasive, Coronary artery bypass grafting, Perioperative, Antibiotics

## Abstract

**Objective:**

To investigate the use of antimicrobials during the perioperative period of minimally invasive coronary artery bypass grafting (MICS CABG) and traditional open-heart bypass grafting. We aimed to determine whether the duration of perioperative antibiotic use and infection rate is significantly different between different surgical methods.

**Methods:**

A total of 471 cases of coronary artery bypass grafting (CABG) were collected from January 2019 to December 2022. Patients were divided into minimally invasive group (229 cases) and a conventional group (242 cases) according to the type of surgery. We compared differences in the duration of antimicrobial use and infection rates between the two groups.

**Results:**

Compared with the conventional group, the minimally invasive group had a significantly shorter average duration of antimicrobial therapy [(1.95 ± 2.40) d vs. (4.67 ± 5.89) d, *P* < 0.001], a higher rate of short antibiotic treatment duration (T ≤ 24 h) [51.97% vs. 7.02%, *P* < 0.001], lower postoperative pneumonia rate [38.86% vs. 56.20%, *P* < 0.001], lower positive rate of blood and surgical site sample culture (1 case and 0 case) vs. (7 cases and 3 cases), *P* < 0.001. Subgroup analysis of different durations of antimicrobial treatment (T ≤ 24 h, 24 h < T ≤ 48 h, and 48 h < T ≤ 96 h) in the minimally invasive group showed that there was no statistically significant difference in the incidence of infection among the various medication durations (*P* > 0.05).

**Conclusion:**

Compared with traditional surgery, MICS CABG requires a significantly shorter duration of perioperative antibiotic treatment duration and a reduced incidence of infection. Extending the duration of antibiotic treatment did not reduce the incidence of infection.

**Trial registration:**

chictr.org.cn ChiCTR2400091571.

## Introduction

Coronary atherosclerotic heart disease (CHD), also known as coronary heart disease, is a disease in which coronary atherosclerosis leads to coronary artery stenosis and insufficient blood supply [1]. CHD causes myocardial ischemia and necrosis, which can lead to loss of heart function. It is estimated that over 125 million people worldwide suffer from CHD, and approximately 35% of people who experience a coronary event in a given year die because of it [2]. Pharmacotherapy, percutaneous coronary intervention (PCI), and CABG are effective methods for treating CHD [3]. Traditional CABG adopts median thoracotomy, which results in relatively large surgical trauma, and some patients are at risk of poor wound healing [4]. The improvement of CABG technology aims to reduce surgical trauma and complications, mainly by reducing incision size and reducing the use of extracorporeal circulation. MICS CABG has developed rapidly in recent years, allowing surgeons to enter the chest through the fourth or fifth intercostal space and greatly reduce incision size [5].

Perioperative infections after cardiac surgery, including surgical site infections, blood infections, pneumonia, and *Clostridium difficile* colitis are reasons for prolonged hospitalization or readmission, mortality, and significantly increased healthcare costs [2, 6]. Perioperative prophylactic use of antibiotics can effectively reduce the incidence of CABG incision infection [7]. Infection of a cardiac surgical site is life-threatening, so clinical physicians often extend the duration of medication. This study retrospectively investigated the use of antibiotics during the perioperative period of CABG, analyzed the medication duration of different incision surgeries, and the incidence of postoperative infections, to provide a basis for rational drug use in clinical practice.

## Methods

### Patients

**Fig. 1:**
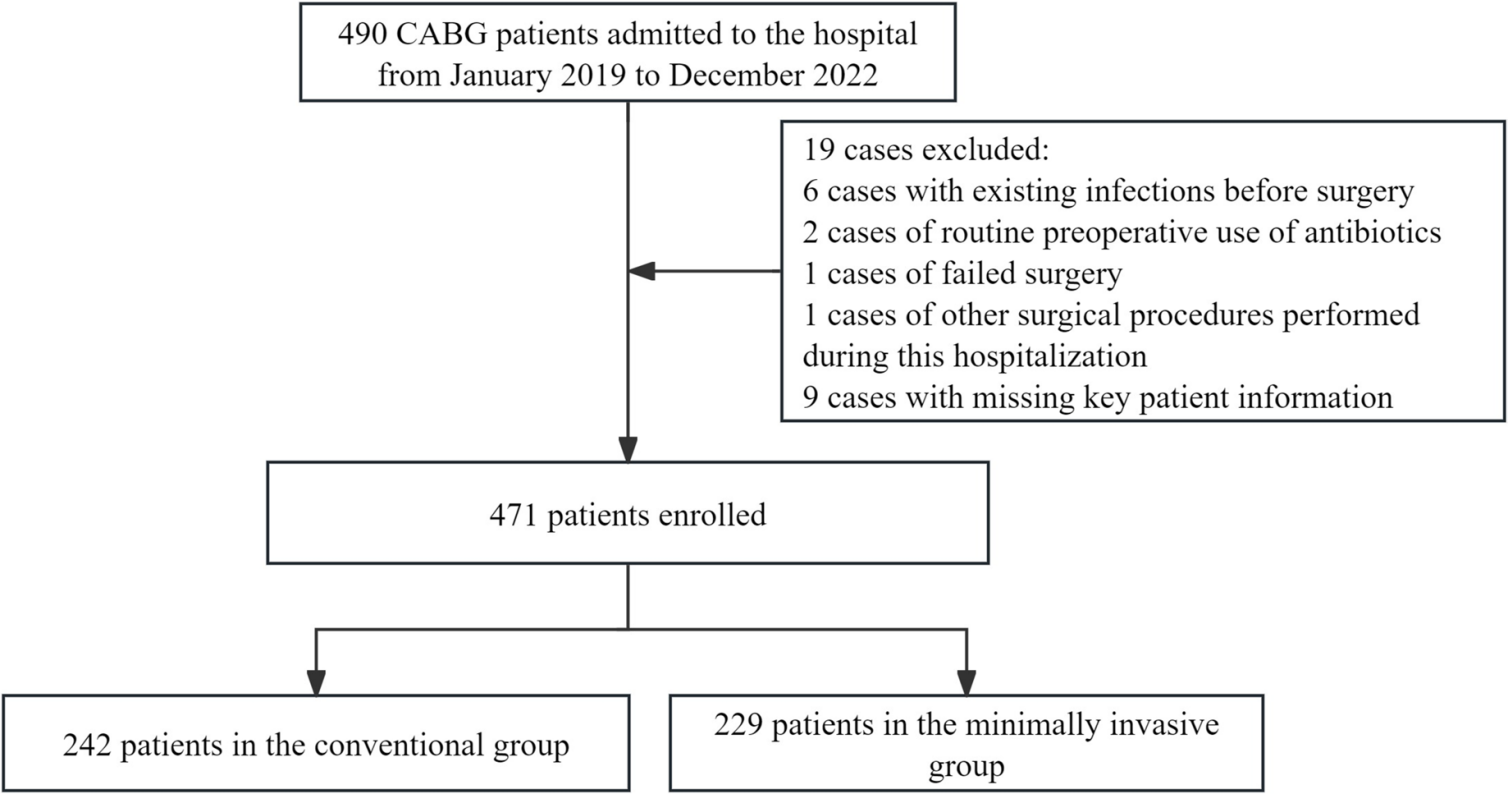
Study flow chart of patient enrollment

CABG patients admitted to the hospital from January 2019 to December 2022 were included. Exclusion criteria were as follows: patients with existing infections before surgery; cases of routine preoperative use of antibiotics; cases of failed surgery; cases of other surgical procedures performed during this hospitalization; cases with missing key patient information. A total of 19 cases were excluded, and 471 patients were ultimately included (Fig. 1).

### Study design

According to the type of surgical incision, the study cases were divided into a minimally invasive group and a conventional group. The minimally invasive group had undergone incision of the 4th or 5th intercostal space of the left anterior chest, with an incision length of approximately 5–7 cm; the conventional group had undergone median thoracotomy, with an incision length of approximately 20–25 cm. Both groups of patients were treated with antibiotics 0.5 to 1 h before surgery. If the surgery time exceeded 3 h, additional medication was administered during the surgery. In some cases, clinicians extended the postoperative antibiotic prophylaxis time or switched to other antibiotics based on the patient’s infection situation. Relevant indicators were extracted through hospitalization medical records. Baseline indicators included gender, age, weight, preoperative hospitalization days, and underlying disease status. The main outcome measures included the antibiotic cost, number of antibiotic types used, antibiotic treatment duration, and infection rate. The secondary outcome measures included surgical duration, intraoperative blood loss, days of ventilator use, days of urinary catheter use, postoperative hospitalization days, total healthcare cost, and total medication cost. All antibiotics used by patients during this hospitalization period were included in the study. Of note, patients experienced varying degrees of fever and elevated blood infection indicators after CABG surgery, and some cases had incomplete disease course records or lacked clinical symptom descriptions. The following objective indicators were used in this study to classify cases as infection cases: cases with positive pneumonia indicators, positive urine culture, positive blood culture, and positive surgical site specimen culture. Positive pneumonia indicators included chest CT/X-ray indicating the presence of pulmonary infection or positive sputum culture. Positive surgical site specimen culture included a positive culture at the tip of the catheter, drainage fluid, wound secretions, and wound swabs. If a patient did not undergo relevant tests, this patient was recorded as a non-infected case.

### Statistical analysis

All data were analyzed using SPSS 25.0 statistical software. Measurement data are expressed as mean ± standard deviation (‾x ± s) and range, using a t-test. Count data are expressed as examples or rates (%), using a χ^2^ -test;* P* < 0.05 indicates statistical significance.

## Results

### Basic information and clinical data

A total of 471 patients were included, including 289 males and 182 females, aged 37 to 87 (67.08 ± 8.37) years old, with a median age of 68 years. There were 242 patients in the conventional group and 229 patients in the minimally invasive group. The baseline data of the two patient groups are shown in Table 1.

**Table 1:** Baseline characteristics of patients in both groups

Variables	Conventional group (*n* = 242)	Minimally invasive group (*n* = 229)	χ^2^/t	*P*
Gender (male)/(*n*, %)	150, 61.98%	139, 60.70%	0.082	0.777
Age/y	64.95 ± 8.58	66.86 ± 9.16	− 2.331	0.02
Weight/kg	66.71 ± 12.58	67.98 ± 12.15	− 1.101	0.271
Preoperative hospitalization days/d	6.99 ± 5.48	6.98 ± 3.42	0.012	0.991
Hypertension/(*n*, %)	139, 57.44%	128, 55.90%	0.114	0.780
Diabetes/(*n*, %)	76, 31.40%	87, 37.99%	2.255	0.146
Cerebrovascular disease/(*n*, %)	48, 19.83%	50, 21.83%	0.285	0.650

### Main outcome measures

The cost of antibiotics, number of antibiotics types, and antibiotic treatment duration in the minimally invasive group were all significantly lower than those in the conventional group (*P* < 0.001). The positive rates of urine culture, pneumonia indicators, blood culture, and surgical site specimen culture in the minimally invasive group were also significantly lower than those in the conventional group (*P* < 0.001). There were 7 positive blood culture cases and 3 positive surgical site specimen culture cases in the conventional group, and 1 positive blood culture case and 0 positive surgical site specimen culture case in the conventional group. The main outcome indicators of the two groups of patients are shown in Table 2.

**Table 2:** Main outcomes of patients in both groups

Variables	Conventional group (*n* = 242)	Minimally invasive group (*n* = 229)	χ^2^/t	*P*
Cost of antibiotics/RMB	456.49 ± 294.3	100.32 ± 103.34	16.455	< 0.001
Number of antibiotic types	1.26 ± 0.57	1 ± 0	6.940	< 0.001
Antibiotics duration (days)	3.65 ± 2.17	1.38 ± 1.2	13.694	< 0.001
Positive cases of pneumonia indicators (*n*, %)	136, 56.20%	89, 38.86%	14.169	< 0.001
Positive cases of urine culture (*n*, %)	7, 2.89%	5, 2.18%	0.238	< 0.001
Positive cases of blood culture and surgical site specimen culture (*n*, %)	23, 9.50%	1, 0.44%	20.004	< 0.001

### Secondary outcome measures

The minimally invasive group had significantly lower surgical duration, intraoperative blood loss, days of ventilator use, days of urinary catheter use, postoperative hospitalization days, total cost, and total medication cost compared to the traditional group (*P* ≤ 0.001). The secondary outcome indicators of the two patient groups are shown in Table 3.

**Table 3:** Secondary outcome indicators for the two patient groups

Variables	conventional group (*n* = 242)	minimally invasive group (*n* = 229)	*t*	*P*
Surgical duration (min)	348 ± 83.21	292.32 ± 93.4	6.611	< 0.001
Intraoperative blood loss (mL)	1218.79 ± 679.29	468.37 ± 224.62	13.894	< 0.001
Duration of mechanical ventilation (days)	0.65 ± 1.19	0.47 ± 0.2	9.386	< 0.001
Duration of urinary catheter(days)	3.14 ± 1.38	2.46 ± 1.04	5.819	< 0.001
Postoperative hospitalization days (days)	8.6 ± 2.64	7.82 ± 2.43	3.224	0.001
Total cost (RMB)	84,061.36 ± 15,854.52	71,617.21 ± 12,883.46	8.686	< 0.001
Total medication cost (RMB)	8379.44 ± 2353.86	6283 ± 2041.26	9.676	< 0.001

### Antibiotics duration

The duration of antimicrobial treatment (T ≤ 24 h) in the minimally invasive group was 51.97%, which was higher than that in the median thoracotomy group, and the difference was statistically significant (*P* < 0.001). There were 84 patients in the minimally invasive group who only received preoperative and intraoperative prophylactic medication, accounting for 36.24%. The antibiotic treatment duration for the two groups is shown in Table 4.

**Table 4:** Antibiotic treatment duration for the two patient groups

Variables	Conventional group (*n*, %)	Minimally invasive group (*n*, %)	χ^2^	*P*
T ≤ 24 h	17, 7.02%	119, 51.97%	115.707	< 0.001
24 h < T ≤ 48 h	62, 25.62%	27, 11.79%	14.683	< 0.001
48 h < T ≤ 96 h	60, 24.79%	56, 24.45%	0.007	1.000
T > 96 h	103, 42.56%	27, 11.79%	55.754	< 0.001

### Antibiotic treatment duration and infection rate of the minimally invasive group

There was no statistically significant difference in the positive rates of urine culture, pneumonia indicators, blood culture, and surgical site specimen culture among different antibiotic treatment duration groups within the minimally invasive group (*P* > 0.05). The infection rates of different antibiotic treatment duration groups in the minimally invasive group are shown in Table 5.

**Table 5:** Antibiotic treatment duration and infection rate for the minimally invasive group

Variables	T ≤ 24 h (*n*, %)	24 h < T ≤ 48 h (*n*, %)	48 h < T ≤ 96 h (*n*, %)	*P*_*12*_	*P*_*23*_	*P*_*13*_
Positive pneumonia indicators	42, 35.29%	7, 25.93%	23, 41.07%	0.379	0.226	0.283
Positive blood culture and surgical site specimen culture	1, 0.84%	0, 0%	0, 0%	1	1	1
Positive urine culture	4, 3.36%	1, 3.70%	0, 0%	1	0.325	0.307

## Discussion

MICS CABG has significant advantages over traditional CABG. Due to the small incision size, minimally invasive surgery reduces incision pain and facilitates faster postoperative recovery. There is also less bleeding and a lower risk of blood transfusion. Furthermore, there is generally no need for extracorporeal circulation when MICS CABG is performed [8, 9]. However, minimally invasive surgery requires physicians to have high-level technical skills and a thorough understanding of the surgical indications. MICS CABG is not suitable for patients with significant enlargement of the heart, arrhythmia, small coronary artery lumen, severe wall sclerosis, or the need for other cardiac surgeries.

Infection of the sternum surgical site is a severe complication of traditional CABG. The incidence of infection in superficial and deep surgical sites ranges from 1.3% to 12.8% [2]. Minimally invasive surgery avoids a midline incision on the sternum, greatly reducing the incidence of surgical site infections. In this study, there was only one patient with positive blood culture in the minimally invasive surgery group and no cases of surgical site infection. There were 23 positive cases of blood culture and surgical site sample culture in the traditional surgical group.

Postoperative pneumonia is a common complication of cardiac surgery. Cardiac surgery generally has a long duration, significant trauma, and painful incisions, leading to difficulty in sputum excretion and respiratory function damage [10, 11]. Perioperative mechanical ventilation and extracorporeal circulation can also cause lung tissue damage. Therefore, pulmonary infection is the most common site of infection after cardiac surgery, accounting for more than half of all infections. In this study, the incidence of postoperative pneumonia in the minimally invasive group was lower than that in the traditional surgical group, but there was no statistically significant difference in the incidence of postoperative pneumonia among patients with different antibiotic treatment durations. In fact, some common features of patients after CABG are consistent with the diagnostic criteria for pneumonia, such as elevated body temperature and blood inflammatory indicators, which may lead to an over-diagnosis of pneumonia. Therefore, physicians should accurately determine whether a pulmonary infection is present and whether antibiotic treatment is needed based on the patient’s clinical symptoms [12]. Some patients in this study showed positive sputum culture or pneumonia on CT examination, which met the diagnostic criteria for pneumonia. However, they only received prophylactic antibiotics for 24 h during the perioperative period, rather than long-term antibiotic treatment. Providing respiratory management measures to these patients often allows them to recover and be discharged. Therefore, long-term and continuous comprehensive infection control can reduce the incidence of postoperative infections. Specific measures include preoperative respiratory training, early weaning, frequent turning and patting, encouraging sputum production, bronchial suction, semi-recumbent position, reducing nasogastric tube retention time, avoiding extracorporeal circulation, avoiding excessive use of acid suppressants, nutritional management, blood sugar control, early mobilization, and reducing hospital stay, among others [13].

Minimally invasive cardiac surgery only requires preoperative and intraoperative prophylactic use of antibiotics. The duration of medication should be 24 h at minimum, not exceeding 48 h [6]. Overextending the duration of medication can increase the risk of bacterial resistance. There is no evidence to suggest that the use of antibiotics after incision closure can reduce the risk of surgical site infection, and antibiotics should be discontinued when the incision is closed [14]. Since cardiac surgery patients may have a variety of risk factors for postoperative infection, including old age, obesity, diabetes, catheter retention, cardiopulmonary bypass, blood transfusion risk and prolonged operation time, physicians in many countries do not strictly follow the antibiotic standard management guidelines, and the phenomenon of prolonged preventive medication is common [15]. One study showed that shortening the perioperative prophylactic antibiotic duration for adult cardiac surgery patients from 56 to 32 h does not increase surgical site infection rates, hospital-acquired infection rates, and mortality rates, but can reduce antibiotic resistance and medical costs [16]. In this study, 119 patients underwent antibiotic treatment for less than 24 h, of which 84 patients received preoperative and intraoperative medication and did not continue antibiotic use after the incision was closed. All 119 patients had no surgical site infection. Therefore, on the premise of improving intraoperative aseptic technology and implementing comprehensive perioperative infection control measures, clinicians should have confidence in the rational use of antibiotics for infection prevention.

## Conclusion

The results of this study show that compared with traditional CABG, MICS CABG can shorten the duration of antibiotic treatment, shorten surgical time, reduce intraoperative bleeding, reduce the number of days of ventilator use and postoperative hospitalization, and lower medical costs. While reducing the use of antibiotics, the incidence of postoperative pneumonia, surgical site infections, and urinary tract infections decreases. The prophylactic use of antibiotics in minimally invasive cardiac surgery is only necessary before and during surgery, and extending antibiotic treatment does not have significant benefits. There are limitations in this study, such as failure to statistically analyze differences in blood infection indicators, failure to consider the number of surgical bypass vessels, failure to consider all underlying diseases, and failure to study readmission and mortality rates.

## Data Availability

The datasets generated and/or analyzed during the current study are available upon request from the corresponding author upon reasonable request.
